# Humanin Restores Metabolic Hormone Homeostasis of Leptin, Ghrelin, Irisin and Asprosin in Streptozotocin-Induced Diabetic Mice

**DOI:** 10.3390/metabo16060373

**Published:** 2026-05-29

**Authors:** Ferah Bulut, Muhammed Adam, Aslısah Ozgen, Mete Ozcan

**Affiliations:** Department of Biophysics, Faculty of Medicine, Firat University, Elazig TR23119, Turkey; ferahbulut94@gmail.com (F.B.); mehmed-adam@hotmail.com (M.A.); aslisahozgen@gmail.com (A.O.)

**Keywords:** humanin, diabetes, asprosin, leptin, ghrelin, irisin

## Abstract

**Objective**: Diabetes mellitus is closely associated with mitochondrial dysfunction, which disrupts cellular energy metabolism and perturbs hormonal homeostasis. Humanin (HN), a 24-amino acid peptide encoded within the mitochondrial genome, has attracted considerable attention due to its cytoprotective and metabolic regulatory properties. Despite its recognized biological potential, the role of HN in coordinating key metabolic hormone networks under diabetic conditions remains poorly understood. This study aimed to investigate the integrative effects of repeated humanin administration on key metabolic hormones leptin, ghrelin, irisin, and asprosin and its potential role in restoring hormonal homeostasis in a streptozotocin (STZ)-induced diabetic mouse model. **Materials and Methods**: Forty male mice were randomly assigned to four groups (*n* = 10 for each group): control, HN (4 mg/kg/day), STZ (150 mg/kg), and STZ + HN. Humanin was administered intraperitoneally for 15 consecutive days. Serum levels of leptin, asprosin, irisin, and ghrelin were measured using enzyme-linked immunosorbent assay (ELISA), and data were analyzed using one-way ANOVA followed by Tukey’s post hoc test. **Results**: STZ-induced diabetes markedly disrupted metabolic hormone balance, as indicated by decreased leptin and irisin levels and increased asprosin concentrations. Repeated HN treatment effectively restored leptin levels and suppressed asprosin concentrations, while irisin levels showed a relative increase compared to the STZ animals. In addition, ghrelin levels were significantly elevated in HN-treated diabetic mice compared to untreated STZ animals. **Conclusions**: These findings indicate that humanin exerts an integrative, multi-hormonal regulatory effect, supporting the restoration of metabolic and endocrine homeostasis under diabetic conditions.

## 1. Introduction

Diabetes mellitus (DM) represents a major global public health challenge. By 2022, an estimated 828 million adults worldwide were living with diabetes, representing an increase of approximately 630 million cases since 1990 [1]. This chronic metabolic disorder is characterized by impaired insulin secretion and/or insulin resistance, leading to systemic complications driven by oxidative stress, inflammation, and apoptosis [2]. Central to DM pathogenesis is mitochondrial dysfunction, which disrupts cellular bioenergetics, increases reactive oxygen species (ROS) production, and contributes to pancreatic β-cell failure. Emerging evidence suggests that mitochondrial-derived peptides (MDPs) act as novel modulators of cellular survival and metabolic homeostasis [3]. Among these, humanin (HN), a 24-amino acid peptide encoded within the mitochondrial genome, has attracted substantial interest due to its cytoprotective, anti-apoptotic, and metabolic regulatory properties. Humanin expression is closely linked to mitochondrial stress responses and cellular adaptive mechanisms under pathological conditions. Previous studies have demonstrated that HN enhances insulin sensitivity, protects β-cell function, attenuates oxidative stress-induced cellular injury, and improves mitochondrial integrity in metabolic disorders [3,4]. Mechanistically, HN inhibits Bax translocation to mitochondria, suppresses caspase-dependent cell death under hyperglycemic conditions, and activates AKT, ERK1/2, and STAT3 signaling via the gp130/WSX-1 receptor complex, thereby intersecting with key pathways involved in energy metabolism, cellular survival, and inflammatory regulation [5]. Furthermore, growing evidence suggests that HN may function as an integrative metabolic regulator by modulating mitochondrial homeostasis, redox balance, and stress-responsive signaling networks. Collectively, these findings position HN as a promising candidate for modulating mitochondrial health and systemic metabolic homeostasis in diabetes.

Leptin, an adipocyte-derived hormone, primarily regulates appetite and energy expenditure through hypothalamic STAT3 signaling [6]. Impaired leptin signaling is closely linked to insulin resistance, mitochondrial dysfunction, and systemic metabolic dysregulation [7,8]. Similarly, ghrelin, a stomach-derived orexigenic peptide, regulates appetite, glucose homeostasis, and energy utilization [9]. Previous studies suggest that ghrelin contributes to cellular energy regulation and mitochondrial bioenergetics under metabolic stress conditions [10,11]. Together, leptin and ghrelin form a coordinated hormonal network involved in appetite regulation and systemic energy metabolism. These findings further suggest a potential association between mitochondrial dysfunction and appetite-regulating hormonal networks under diabetic conditions.

Irisin, an adipomyokine released from skeletal muscle during exercise, promotes the browning of white adipose tissue in a PGC-1α-dependent manner and enhances insulin sensitivity via AMPK and PI3K/AKT pathway [12,13]. Previous studies also suggest that irisin contributes to mitochondrial function and cellular energy metabolism by promoting mitochondrial biogenesis and reducing oxidative stress [14,15].

Conversely, asprosin, a fasting-induced hepatokine, stimulates hepatic glucose production and is elevated in diabetic states, thereby exacerbating hyperglycemia [16]. Previous studies suggest that asprosin is associated with metabolic dysfunction, inflammation, oxidative stress, and altered mitochondrial activity under diabetic conditions [17]. 

In experimental models, elucidating the pathophysiological mechanisms of diabetes is critical. Streptozotocin (STZ) is widely employed to induce diabetes by causing selective toxicity to pancreatic β-cells through oxidative stress, DNA damage, and mitochondrial dysfunction [18]. Consequently, STZ-induced diabetes models constitute a robust experimental platform for investigating cellular damage and evaluating the therapeutic efficacy of novel metabolic regulators.

Taken together, the dysregulation of key metabolic hormones in diabetes, including decreased leptin and irisin and elevated ghrelin and asprosin, is associated with impaired systemic energy balance and has been linked to mitochondrial dysfunction in previous studies [19,20]. However, the coordinated effects of HN on peripheral metabolic regulation under diabetic conditions remain largely unexplored. Given the established roles of HN in preserving mitochondrial integrity, enhancing insulin signaling, and modulating stress-responsive pathways, HN represents a promising candidate for improving metabolic homeostasis. Therefore, the present study aimed to investigate the effects of HN administration on circulating levels of irisin, asprosin, leptin, and ghrelin in streptozotocin-induced diabetic mice.

## 2. Materials and Methods

### 2.1. Animals and Ethical Approval

A total of 40 adult male BALB/c mice (6–8 weeks old, weighing 25–30 g) were used in this study. The sample size was determined based on previous similar experimental studies using STZ-induced diabetic mouse models. Animals were randomly allocated to experimental groups using a computer-generated randomization method based on body weight. No statistically significant differences were observed among groups in baseline body weights (*p* > 0.05).

The animals were acclimatized for one week prior to the experimental procedures. They were housed under controlled environmental conditions (21 ± 1 °C; 60 ± 5% humidity) with a 12 h light/dark cycle and had ad libitum access to standard laboratory chow and water.

All in vivo experiments were conducted at the Fırat University Experimental Animals Research Center (FUDAM). The study protocol was approved by the Fırat University Animal Experiments Local Ethics Committee (Approval date: 9 October 2019; Protocol No: 2019/47; Decision No: 2019/19). Animals were divided into four experimental groups (n = 10 per group): Control, HN, STZ, and STZ + HN.

### 2.2. Body Weight and Experimental Design

Twenty healthy mice were randomly assigned to control and HN groups (n = 10 in each group). Control animals received daily intraperitoneal injections of phosphate-buffered saline (PBS) (Sigma Aldrich, St. Louis, MO, USA, Product No: P4417) in volumes comparable to those administered to the HN-treated groups throughout the experimental period. The HN group received intraperitoneal HN at a dose of 4 mg/kg once daily for 15 days. The HN used in the study (Catalog No: AS-60886) was obtained from AnaSpec Inc. (Fremont, CA, USA). Before administration, HN was dissolved in PBS. The groups receiving HN treatment received daily intraperitoneal injections of HN between 09:00 and 10:00 for 15 days.

The remaining 20 mice were induced with diabetes as described below. After confirmation of diabetes, the animals were randomly assigned to STZ and STZ + HN groups (n = 10 in each group). The STZ + HN group received intraperitoneal HN (4 mg/kg/day) for 15 days, while the STZ group received daily intraperitoneal injections of PBS in volumes comparable to those administered to the STZ + HN group. The body weight of each mouse was measured at the start of the experiment (Day 1), and the last measurement was taken on the last day of treatment (Day 15). To ensure consistency, measurements were taken at the same time on the indicated days using a digital electronic balance (with 0.01 g accuracy). This data was used to evaluate STZ-induced diabetes and the systemic metabolic effects of HN treatment.

### 2.3. Streptozotocin-Induced Diabetes Model in Mice

STZ was freshly dissolved in 0.1 M sodium citrate buffer (pH 4.5) and administered as a single intraperitoneal injection at a dose of 150 mg/kg. Blood glucose levels were tested after 72 h using a manual glucometer (Optima, Taipei, Taiwan) (all measurements were taken between 9:00 and 10:00 AM). Animals were fasted for 8–10 h prior to measurement to assess fasting blood glucose levels. Mice with fasting blood glucose levels above 250 mg/dL were considered diabetic [21]. The STZ-induced diabetic group was randomly divided into two subgroups: STZ (diabetic control) and STZ + HN (n = 10 in each group). The STZ group received no treatment, while the STZ + HN group received intraperitoneal HN (4 mg/kg) for 15 days.

### 2.4. Measurement of Leptin, Ghrelin, İrisin and Asprosin Concentrations

**Figure 1:**
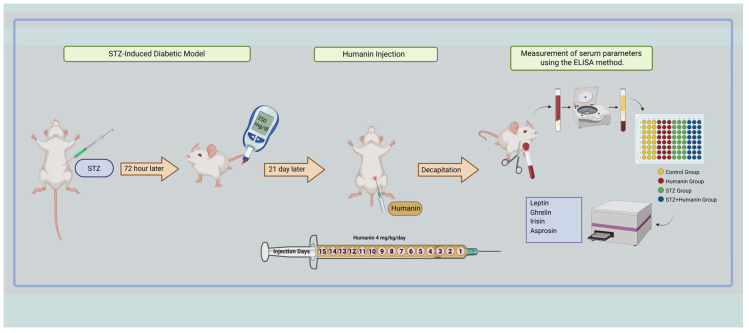
Flowchart of experimental design.

Sixty minutes after the last injection, the animals were euthanized by decapitation, and blood samples were collected immediately. Blood samples were centrifuged at 3500 rpm for 10 min at 4 °C to obtain serum. Serum samples were compartmentalized and stored at −80 °C until analysis. Serum concentrations of leptin, ghrelin, and irisin were measured using an enzyme-linked immunosorbent assay (ELISA) (Elabscience Company, Houston, TX, USA). The leptin ELISA kit had a test range of 0.31–20 ng/mL and a sensitivity of 0.19 ng/mL (catalog no: E-EL-M3008); the ghrelin ELISA kit had a test range of 0.16–10 ng/mL and a sensitivity of 0.1 ng/mL (catalog no: E-EL-M0551). The irisin ELISA kit had a test range of 62.5–4000 pg/mL and a sensitivity of 37.5 pg/mL (catalog no: E-EL-M0392). Serum asprosin concentrations were measured using the ELISA method (USCN catalog number: SEA332Mu). The asprosin ELISA kit had a test range of 1.5–400 ng/mL and a sensitivity of 0.127 ng/mL. Absorbance values were measured using a microplate reader (Figure 1).

### 2.5. Statistical Analyses

Statistical analyses were performed using GraphPad Prism version 11.0.0 (GraphPad Software, San Diego, CA, USA). The normality of data distribution was assessed using the Shapiro–Wilk test. For comparisons among multiple groups, one-way analysis of variance (ANOVA) was used, followed by Tukey’s post hoc test for pairwise comparisons. Data are presented as mean ± standard deviation (SD). A *p*-value of <0.05 was considered statistically significant. Figures were prepared using BioRender.com.

## 3. Results

### 3.1. The Effect of Humanin Application on Body Weight

**Figure 2:**
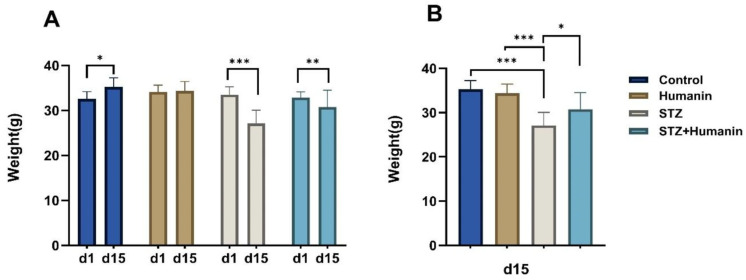
The effect of humanin application on body weight (n = 10 for each group, * *p* < 0.05 compared with control d1 vs. d15 (**A**), *** *p* < 0.001 compared with STZ d1 vs. d15 (**A**), ** *p* < 0.01 compared with STZ + humanin d1 vs. d15 (**A**), * *p* < 0.05 compared with STZ d15 vs. STZ + humanin d15 (**B**), *** *p* < 0.001 compared with STZ d15 vs. STZ + humanin d15 (**B**), one-way analysis of variance followed by a post hoc Tukey HSD test).

The body weight data measured on day 1 were similar for all groups. However, a statistically significant difference was observed between the body weights of the groups during the 15-day experiment. In the control group, a statistically significant increase was observed when comparing weight measurements on days 1 and 15 (*p* < 0.05, Figure 2A). In the HN group, no statistically significant difference was observed between the 1st and 15 days of application (*p* > 0.05, Figure 2A). In the STZ group, when comparing data from days 1 and 15, it was observed that STZ-induced diabetes caused a significant decrease in body weight (*p* < 0.001, Figure 2A). However, when comparing data from the STZ group and the STZ + HN group on day 15, it was observed that HN treatment significantly increased body weight (*p* < 0.05, Figure 2B).

### 3.2. Effects of Administration on Serum Leptin Levels in STZ-Induced Diabetic Mice

**Figure 3:**
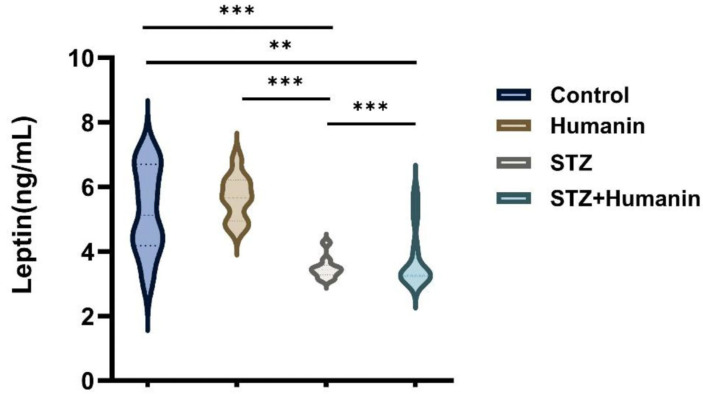
Effects of humanin administration on serum leptin level in STZ-induced diabetic mice (n = 10 for each group, *** *p* < 0.001 compared with control vs. STZ, ** *p* < 0.01 compared with control vs. STZ + humanin, *** *p* < 0.001 compared with humanin vs. STZ, *** *p* < 0.001 compared with STZ vs. STZ + humanin, one-way analysis of variance followed by a post hoc Tukey HSD test).

Serum leptin concentrations differed significantly between groups (*p* < 0.001; Figure 3). STZ-induced diabetes resulted in a marked decrease in leptin levels compared to the control (*p* < 0.001) and HN groups (*p* < 0.01). HN treatment in diabetic mice significantly increased leptin levels compared to the STZ group (*p* < 0.001). However, leptin concentrations in the STZ + HN group remained significantly lower compared to the control group (*p* < 0.001).

### 3.3. Effects of Humanin Administration on Serum Ghrelin Levels in STZ-Induced Diabetic Mice

**Figure 4:**
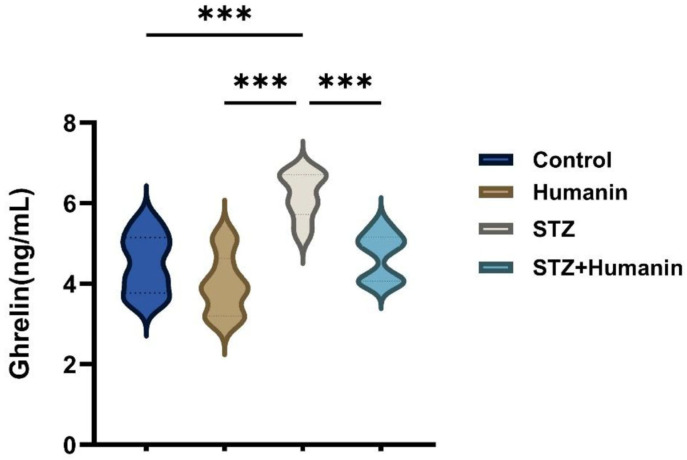
Effects of humanin administration on serum ghrelin level in STZ-induced diabetic mice (n = 10 for each group, *** *p* < 0.001 compared with STZ vs. control group, *** *p* < 0.001 compared with humanin vs. STZ, *** *p* < 0.001 compared with STZ vs. STZ + humanin group, one-way analysis of variance followed by a post hoc Tukey HSD test).

A significant difference was observed in serum ghrelin levels between the groups. Ghrelin concentrations were significantly higher in the STZ + HN group compared to the STZ group (*p* < 0.001, Figure 4). The STZ-induced diabetes group showed a statistically significant increase when compared to the control group (*p* < 0.001). Furthermore, a significant difference was observed between the HN group and the STZ group (*p* < 0.001).

### 3.4. Effects of Humanin Administration on Serum Irisin Levels in STZ-Induced Diabetic Mice

**Figure 5:**
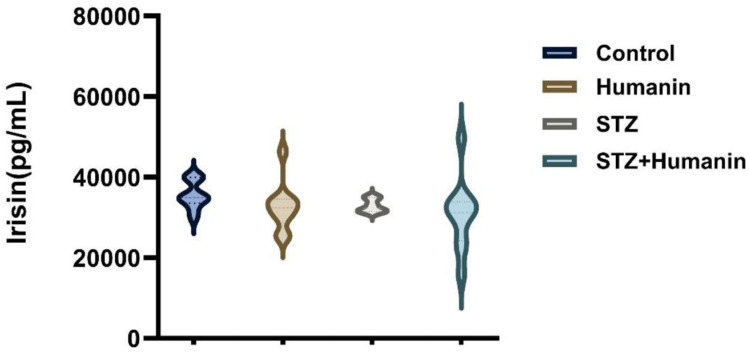
Effects of humanin administration on serum irisin level in STZ-induced diabetic mice (n = 10 for each group, no significant difference was found between the groups, one-way analysis of variance followed by a post hoc Tukey HSD test).

Mean serum irisin levels were relatively higher in the STZ + HN group compared to the STZ group. In addition, HN-treated groups showed broader value distributions, whereas the STZ group exhibited a narrower distribution pattern. However, despite these observational differences, the variation among groups did not reach statistical significance (*p* > 0.05; Figure 5).

### 3.5. Effects of Humanin Administration on Serum Asprosin Levels in STZ-Induced Diabetic Mice

**Figure 6:**
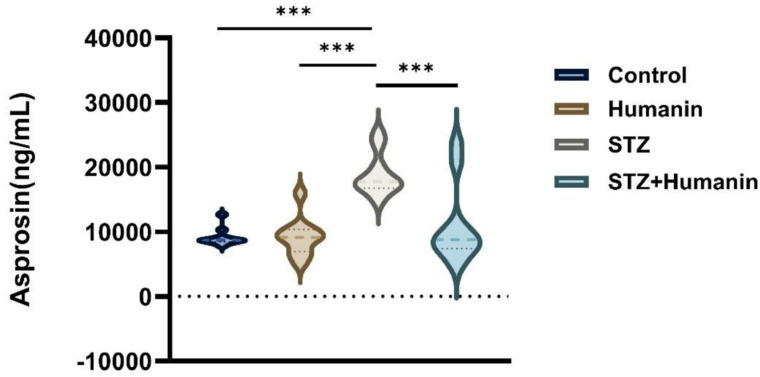
Effects of humanin administration on serum asprosin level in STZ-induced diabetic mice (n = 10 for each group, *** *p* < 0.001 compared with control vs. STZ, *** *p* < 0.001 compared with humanin vs. STZ, *** *p* < 0.001 compared with STZ vs. STZ + humanin, one-way analysis of variance followed by a post hoc Tukey HSD test).

Serum asprosin concentrations differed significantly among groups (*p* < 0.001; Figure 6). STZ administration significantly increased circulating asprosin levels compared with both the control and HN groups (*p* < 0.001). HN treatment significantly reduced asprosin levels in diabetic mice (*p* < 0.001). Additionally, a statistically significant difference was observed between the control and HN groups (*p* < 0.001).

## 4. Discussion

The present findings demonstrate that repeated HN administration for 15 days partially improved the dysregulated metabolic hormone profile observed in STZ-induced diabetic mice, particularly through the restoration of leptin levels, suppression of asprosin, and attenuation of diabetes-associated body weight loss. In addition, HN treatment was associated with a non-significant tendency toward increased irisin levels, highlighting the potential of HN as a multifaceted modulator of metabolic homeostasis.

Body weight is a significant indicator of overall metabolic status, and marked weight loss, particularly observed in STZ-induced diabetes models, is a characteristic finding of the disease. In this study, the significant weight loss observed in the STZ group between days 1 and 15 can be explained by catabolic processes developing due to insulin deficiency. In cases of insufficient insulin signaling, the inability of cells to effectively utilize glucose directs the organism towards alternative energy sources; this results in increased mobilization of lipid stores and protein breakdown, leading to a negative energy balance [22]. Our findings show that repeated HN administration for 15 days significantly attenuated diabetes-related body weight loss at day 15. This protective effect of HN on body weight may reflect improved energy utilization together with partial suppression of catabolic processes. These findings further indicate that HN may contribute to the restoration of metabolic balance under diabetic conditions.

Leptin and ghrelin are well-known regulators of energy homeostasis with opposing physiological effects [23,24,25]. In STZ-induced diabetes models, leptin levels are typically reduced, contributing to impaired metabolic homeostasis and increased metabolic stress [23]. In contrast, ghrelin primarily regulates energy intake and glucose homeostasis [24,25]. These two hormones exert opposing effects on energy balance, and leptin deficiency is often associated with elevated ghrelin levels [26,27]. Consistent with these reports, our findings demonstrate that STZ-treated mice exhibited decreased leptin levels alongside increased ghrelin levels, indicating a disruption in metabolic homeostasis. Notably, repeated HN administration attenuated this hormonal imbalance by restoring leptin levels and reducing the compensatory increase in ghrelin. These results support the concept that HN regulates energy homeostasis through coordinated modulation of multiple hormonal pathways rather than a single endocrine axis [4].

Irisin is a myokine secreted by muscles and adipose tissue during exercise that increases energy expenditure and stimulates the browning of white adipose tissue [12,13]. In STZ-induced diabetes models, irisin levels are frequently reported to be decreased or dysregulated [28]. Consistent with the literature, our study found that serum irisin concentrations in the STZ group showed a decreasing trend compared to the control group. Although this variation did not reach the threshold of statistical significance, this observed decrease is consistent with findings in the literature studies showing impaired energy metabolism, mitochondrial dysfunction, and increased metabolic stress in the diabetic process [29]. HN is considered a peptide that reduces mitochondrial stress and improves cellular energy balance [4], and it partially compensated for the decrease in irisin caused by STZ induction and showed a relative increase in irisin levels, although this was not statistically significant. This finding suggests that HN may indirectly improve irisin production by supporting mitochondrial function and energy homeostasis.

Asprosin is a fasting-induced hormone secreted from white adipose tissue that stimulates hepatic glucose production [30,31]. In diabetic models, elevated asprosin levels have been linked to hyperglycemia and increased gluconeogenesis [30]. In this study, STZ-induced diabetic mice showed markedly elevated asprosin levels, which were significantly suppressed by HN treatment. This observation suggests that HN may reduce gluconeogenic responses and improve glucose homeostasis. Furthermore, HN has been shown to support mitochondrial function, decrease oxidative stress, and optimize energy metabolism [3]. Thus, the correction of asprosin imbalance by HN aligns with its systemic metabolic regulatory capacity.

In our study, the regulatory effect of HN on leptin, ghrelin, irisin, and asprosin in STZ-induced diabetes demonstrates that this peptide functions as a central regulator restoring disrupted metabolic balance. In our group’s previously published study, HN administration was reported to stabilize high blood glucose levels and significantly improve hyperglycemic status in diabetic models [32]. The study also demonstrated that HN effectively reversed cellular damage by normalizing STZ-induced oxidative stress markers (TAS, TOS, MDA, and SOD) [32,33]. The improvement in hyperglycemic status induced by humanin is consistent with our current findings, particularly the improvements observed in diabetes-associated body weight regulation and the normalization of metabolic hormone levels. These pathways have also been associated with the regulation of metabolic hormones and cellular energy homeostasis [34].

Mitochondrial dysfunction is a central feature of metabolic disorders, particularly in diabetes mellitus, where impaired oxidative phosphorylation, increased ROS production, and disrupted energy-sensing pathways contribute to systemic metabolic imbalance [20,35]. In this context, mitochondrial-derived peptides such as HN have attracted attention because of their reported roles in cellular stress responses and metabolic regulation [36]. Previous studies suggest that HN may exert cytoprotective effects by preserving mitochondrial integrity, enhancing antioxidant defenses, and modulating signaling pathways including AMPK, PI3K/Akt, and STAT3 [5]. These pathways have also been associated with the regulation of metabolic hormones and cellular energy homeostasis [33]. Although mitochondrial function was not directly evaluated in the present study, the observed alterations in leptin, ghrelin, irisin, and asprosin levels may be indirectly associated with improvements in metabolic and cellular stress responses following HN treatment.

Despite the significant findings of the present study, certain limitations should be acknowledged. Although our group previously demonstrated that HN administration significantly improved hyperglycemia and oxidative stress parameters in STZ-induced diabetic models [32], blood glucose and insulin levels were not evaluated concurrently with metabolic hormone analyses in the present study. Simultaneous assessment of these parameters could have provided additional mechanistic insight into the relationship between HN-mediated hormonal regulation and glycemic control. Furthermore, direct analyses of mitochondrial signaling pathways were not performed and should be investigated in future studies.

From a translational perspective, the ability of HN to modulate multiple metabolic hormones simultaneously may represent a potentially advantageous therapeutic approach in diabetes, where complex endocrine and metabolic dysregulation coexist. In particular, the observed effects on leptin, ghrelin, and asprosin regulation suggest that HN may contribute to the improvement of systemic metabolic balance beyond single-target interventions. However, the present findings are limited to an experimental STZ-induced diabetic mouse model, and the precise molecular mechanisms underlying these effects remain incompletely understood. Therefore, further experimental and clinical studies are needed to evaluate the long-term efficacy, safety profile, optimal dosing strategies, and translational applicability of HN in human metabolic disorders.

## 5. Conclusions

In line with this framework, the observed modulation of leptin, ghrelin, irisin, and asprosin following repeated HN administration suggests that HN may contribute to the restoration of metabolic balance under diabetic conditions. Given the reported association between HN and mitochondrial stress responses in previous studies, these hormonal alterations may be indirectly linked to improvements in cellular energy homeostasis and stress-responsive signaling pathways. Collectively, these findings support the notion that HN functions as a potential integrative regulator of metabolic hormone networks rather than acting through a single endocrine axis.

## Data Availability

The raw data supporting the conclusions of this article will be made available by the authors on request.
